# The analysis of expression of p16 protein in group of 53 patients treated for sinonasal inverted papilloma^☆^

**DOI:** 10.1016/j.bjorl.2017.03.011

**Published:** 2017-04-19

**Authors:** Roland Zydroń, Andrzej Marszałek, Magdalena Bodnar, Paweł Kosikowski, Grażyna Greczka, Małgorzata Wierzbicka

**Affiliations:** a Poznan University of Medical Sciences, Department of Otolaryngology and Oncological Laryngology, Poznan, Poland; b Nicolaus Copernicus University in Torun, Collegium Medicum, Department of Clinical Pathomorphology, Bydgoszcz, Poland; c Poznan University of Medical Sciences & Greater Poland Cancer Center, Oncologic Pathology and Prophylaxis Department, Poznan, Poland; d Poznan University of Medical Sciences, Department of Clinical Patomorphology, Poznan, Poland

**Keywords:** Inverted papilloma, Sinonasal tumors, p16, HPV, Malignant transformation, Recurrences, Papiloma invertido, Tumores nasosinusais, p16, HPV, Transformação maligna, Recorrências

## Abstract

**Introduction:**

Sinonasal inverted papilloma constitute relevant therapeutic problem due to destructive character of growth, tendency to recur and the possibility of malignant transformation. Therefore, many attempts to identify risk factors for inverted papilloma occurrence have been undertaken, as well as research to find markers that would allow for the earlier detection of tumors and the application of adequate therapy. A widely known risk factor of inverted papilloma is HPV infection. One of the markers of HPV infection and the ongoing effect of this change (although arousing some controversy) is the expression of the p16 protein.

**Objective:**

The aim of the study was to analyze the correlation between the expression of p16 as a surrogate of HPV infection in analyzed histopathological material and epidemiological variables, recurrences or malignant transformation.

**Methods:**

The retrospective study includes a group of 53 patients (18 women and 35 men) undergoing treatment for sinonasal inverted papilloma in the period of 2002–2012. The intensity of the p16 protein in histopathological material was scored as: 0 – no expression, 1 – diffuse expression (borderline) and 2 – positive expression; or 0 – no expression/diffuse expression (borderline); 1 – positive expression. The Ethics Committee agreement was obtained (1089/12; 245/13).

**Results and conclusion:**

There was no statistically significant relationship between the expression of p16 and the age of patients, cigarette smoking, tumor location, tumor staging according to the Krouse and Cannady classification, the presence of dysplasia or the occurrence of relapse.

## Resumo

**Introdução:**

Papiloma invertido nasossinusal constitui um problema terapêutico relevante devido ao caráter destrutivo do crescimento, a tendência à recorrência e a possibilidade de transformação maligna. Assim, muitas tentativas têm sido realizadas para identificar fatores de risco para ocorrência de papiloma invertido, bem como pesquisas para encontrar marcadores que permitam a detecção precoce de tumores e a utilização de terapia adequada. Um fator de risco amplamente conhecido de papiloma invertido é a infecção pelo HPV. Um dos marcadores da infecção por HPV e do efeito contínuo dessa alteração (embora suscitando alguma controvérsia) é a expressão da proteína p16.

**Objetivo:**

Analisar a correlação entre a expressão de p16 como um substituto da infecção pelo HPV no material histopatológico analisado e as variáveis epidemiológicas, recorrências, ou transformação maligna.

**Método:**

O estudo retrospectivo inclui um grupo de 53 pacientes (18 mulheres e 35 homens) submetidos a tratamento para papiloma invertido nasossinusal no período de 2002 a 2012. A intensidade da expressão da proteína p16 no material histopatológico foi pontuada como: 0 - sem expressão, 1 - expressão difusa (limite) e 2 - expressão positiva; ou 0 - sem expressão/expressão difusa (limite); 1 - expressão positiva. O Comitê de Ética aprovou o estudo (1089/12; 245/13).

**Resultados e conclusão:**

Não houve relação estatisticamente significante entre a expressão de p16 e a idade dos pacientes, o tabagismo, a localização tumoral, e o estadiamento tumoral de acordo com a classificação de Krouse e Cannady, presença de displasia ou ocorrência de recidiva.

## Introduction

Sinonasal Inverted Papillomas (IPs) develop from the ciliated epithelium covering the nasal cavity and paranasal sinuses, called Schneiderian membrane. They constitute 0.4%–4.7% of sinonasal tumors.^1^ The Schneiderian membrane is an epithelial lining similar in structure to the respiratory epithelium of the lower respiratory tract, but unlike that tissue, is derived from the ectoderm not the endoderm. Schneiderian epithelium is composed of a single layer of cylindrical ciliated cells, with a small amount of goblet cells. IPs are built with hyperplastic epithelium which grows endophytically into down-lying stroma. The epithelial growths are limited by the basement membrane. The epithelium forming the IP is formed from a few to several layers of cells of the type of squamous, transitional and/or ciliated columnar epithelium. Usually, an admixture of goblet cells is also visible. IP is characterized by a low number of mitoses (mainly located close to the base). Sometimes, superficial keratosis (10%–20% of cases) and intraepithelial dysplasia (5%–10% of cases) is observed.^2^

The most well-known IP risk factor is HPV infection.^3^, ^4^, ^5^, ^6^ The other considered risk factors for IP development are: inflammatory infiltration,^7^ welding fumes and organic solvents.^8^

The maintenance of the transformed phenotype, dependent upon high-risk human papillomavirus (hrHPV) infection, is mainly caused by the expression of two viral oncogenes, E6 and E7. hrHPV E6 protein initiates carcinogenesis by its ability to target p53. The E6/UBE3A (E6-AP) ubiquitin ligase complex targets TP53 for ubiquitylation and proteasomal degradation. Moreover, hrHPV E6 activates telomerase (TERT) to regulate cellular adhesion, and uncontrolled proliferation.^9^

The hrHPV E7 protein initiates carcinogenesis by degradation and inactivation of the retinoblastoma tumor suppressor protein (RB1) by preventing it from binding to the transcription factor E2F, and thereby promoting cell cycle progression.^10^ E2F activation by hrHPV E7 is thought to stimulate expression of the tumor suppressor p16 [INK4A]. The p16 [INK4A] tumor suppressor inhibits CDK4/CDK6 activity by recalling the complex formation with D-type cyclins (CCND). CDK4/6 inhibition causes accumulation of hypophosphorylated, active, RB1 tumor suppressor, which triggers G_1_ cell cycle arrest by repressor complex formation with E2F/DP transcription factors.^10^

Recent studies suggest that one of the main carcinogenic markers/determinants of the degradation of RB1 is caused by high-risk HPV E7 is p16INK4A expression, whereas low-risk HPV E7 proteins do not trigger p16INK4A.^11^

A general screening approach consistent with the finding that p16INK4A expression is a biomarker specifically for high-risk HPV-infected lesions and cancers are the evaluation of protein products of the p16INK4A tumor suppressor gene also referred to as p16.

The aim of the study was to analyze the correlation between the expression of p16 as a surrogate of HPV infection in analyzed histopathological material and epidemiological variables, recurrences or malignant transformation in a group of 53 individuals treated for IP.

## Methods

The retrospective study includes a group of 53 patients (18 women and 35 men) undergoing treatment for sinonasal IP in the period of 2002–2012 in the single institution.

The histopathological examinations were performed by three experienced pathologists and consensus to the way of describing of evaluations was determined before the beginning of the study.

Paraffin blocks were cut on a manual rotary microtome (AccuCut, Sakura, Torrance, USA) into 4 μm thick paraffin sections, and placed on extra adhesive slides (SuperFrostPlus, MenzelGlasser, Braunschweig, Germany). The immunohistochemical procedure was standardized using a series of positive and negative control reactions on formalin-fixed paraffin-embedded tissue sections.

The immunohistochemical staining was performed using automated slide-processing system Benchmark GX Platform (Ventana Medical Systems, Tuscon, AZ, USA) with primary mouse monoclonal antibody CINtec^®^ p16 antibody (clone E6H4™, cat. n° 705-4713; Ventana Medical Systems, Tuscon, AZ, USA), and visualization system UltraView DAB IHC Detection Kit (Ventana Medical Systems, Tuscon, AZ, USA) recommended by the manufacturer. Finally, the slides were dehydrated, cleared in series of xylenes, and coverslipped with Tissu-Tek (Sakura, Japan).

The pathologists evaluating the immunohistochemical expression of the examined antigens worked independently, and were blinded to the patients’ clinical and other data. The intensity of the p16 protein was scored as: 0 – no expression, 1 – diffuse expression (borderline), and 2 – positive expression; or 0 – no expression/diffuse expression (borderline); 1 – positive expression.

The Ethics Committee agreement was obtained (1089/12; 245/13).

## Results

The age of patients ranged from 29 to 80 years; average: 53.72 years (women 40–75 years, average: 58.2 years; men 32–80 years, average: 51.4 years). There was no statistically significant correlation between the age of patients and the expression of p16 (*p* = 0.3015) (Table 1).

**Table 1:** Sex, smoking, duration of symptoms and the age of patients in p16 positive and negative group.

Number of patients	p16− (NEG)		p16+(POS)		Value of the test		*p*
Sex	W	M	W	M	Chi^2^(1) = 1.15		0.2835
	13	20	5	15			
Smoking	Yes	No	Yes	No	Chi^2^(1) = 0.061		0.8049
	16	17	9	11			
Duration of symptoms	Below average	Above average	Below average	Above average	T(51) = 0.221493		0.825593
	24	9	12	8			
Age	Below average	Above average	Below average	Above average	T(51) = 1.112518		0.271132
	17	16	9	11			

Number of patients	p16− (NEG)		p16 borderline (NEG)		p16+ (POS)		*p*
Sex	W	M	W	M	W	M	0.55787
	3	5	10	15	5	15	
Smoking	Yes	No	Yes	No	Yes	No	0.96541
	4	4	12	13	9	11	
Duration of symptoms	Below average	Above average	Below average	Above average	Below average	Above average	0.4817
	6	2	15	10	12	8	
Age	Below average	Above average	Below average	Above average	Below average	Above average	0.3015
	6	2	9	16	9	11	

Average duration of symptoms was 17.6 months (0–120 months). In 75% of cases, duration was less than 24 months (Table 1).

In the analyzed group, the percentage of patients with the full expression of p16 was 37.64% and 84.91%, also taking into account borderline expression.

Twenty-five (47.16%) patients stated a history of cigarette smoking. The percentage of smokers in the group of patients with p16 expression and in the group of patients expressing diffuse expression/absent expression was similar (45% vs. 48%). There was no statistically significant correlation between smoking and p16 protein expression (Table 1).

There was no statistically significant correlation between the location of the tumor and the expression of p16; however, the percentage of patients with full expression of p16 was higher in the case of changes spreading beyond the nasal cavity, and hence, was more advanced (Table 2).

**Table 2:** Location of the tumor in p16 positive and negative group.

Number of patients	Nasal cavity	Nasal cavity + maxillary sinus	Nasal cavity + ethymoid	Other localizations	Value of the test	*p*
p16− (NEG)	8 (15.09%)	7 (13.21%)	8 (15.09%)	10 (18.87%)	Chi^2^(3) = 0.763	0.85823
p16+ (POS)	3 (5.66%)	4 (7.55%)	6 (11.32%)	7 (13.21%)		
p16− (NEG)	1 (1.89%)	1 (1.89%)	1 (1.89%)	5 (9.43%)		0.44422
p16 borderline (NEG)	7 (13.21%)	6 (11.32%)	7 (13.21%)	5 (9.43%)		
p16+ (POS)	3 (5.66%)	4 (7.55%)	6 (11.32%)	7 (13.21%)		

There was no statistically significant correlation between the tumor staging according to Krouse^12^ classification and the expression of p16 (Table 3). Nevertheless, the proportion of p16+ patients is higher in the group of patients with T2 (56%) and T3 (39%) tumors than in patients with early changes (T1 – 27%). Low percentage (20%) of p16+ patients in the T4 group may result from it being a small group (*n* = 5).

**Table 3:** Staging according to Krouse classification in p16 positive and negative group.

Number of patients	T1	T2	T3	T4	Value of the test	*p*
p16− (NEG)	8 (15.09%)	4(7.55%)	17 (32.08%)	4 (7.55%)	Chi^2^(3) = 2.427	0.48865
p16+ (POS)	3 (5.66%)	5(9.43%)	11 (20.75%)	1 (1.89%)		
p16− (NEG)	1 (1.89%)	1 (1.89%)	4 (7.55%)	2 (3.77%)		0.54503
p16 borderline (NEG)	7 (13.21%)	3 (5.66%)	13 (24.53%)	2 (3.77%)		
p16+ (POS)	3 (5.66%)	5 (9.43%)	11 (20.75%)	1 (1.89%)		

According to the Cannady classification,^13^ 20 (37.74%) patients had a tumor classified as “A”, 28 (52.83%) patients were “B”, and 3 (5.66%) were “C”. The percentage of patients showing full expression of the p16 protein in each group was 40%, 34% and 33%, respectively.

There was no statistically significant correlation between the occurrence of dysplasia and the expression of p16. The percentage of patients with dysplasia in the p16+ group was 45% (Table 4).

**Table 4:** Occurrence of dysplasia in p16 positive and negative group.

Dysplasia degree	Without	I	II	III	Value of the test	*p*
p16− (NEG)	21 (39.62%)	9 (16.98%)	1 (1.89%)	2 (3.77%)	Chi^2^(3) = 6.944	0.07371
p16+ (POS)	11 (20.75%)	4 (7.55%)	5 (9.43%)	0 (0.0%)		
Whole	32 (60.38%)	13 (24.53%)	6 (11.32%)	2 (3.77%)		

Dysplasia degree	Without	I	II	III	Razem	*p*
p16− (NEG)	4 (7.55%)	2 (3.77%)	1 (1.89%)	1 (1.89%)	8 (15.09%)	0.15801
p16 borderline (NEG)	17 (32.08%)	7 (13.21%)	0 (1.89%)	1 (1.89%)	25 (47.17%)	
p16+ (POS)	11 (20.75%)	4 (7.55%)	5 (9.43%)	0 (0.0%)	20 (37.74%)	
Whole	32 (60.38%)	13 (24.53%)	6 (11.32%)	2 (3.77%)	53 (100%)	

In 5 (9.43%) patients, transformation to squamous cell carcinoma was observed. In this group, one patient had full expression of p16, two patients had borderline expression and the final two had a total lack of p16 expression.

The average follow-up time was 51 months (4.5 months – 154 months). Every patient was referred to regular check-up visits. 12 of them (22.6%) did not respond to referral.

Recurrence occurred in 25 (47.17%) patients. The average time to recurrence was 36.9 months (2.6 months – 9.6 years).The percentage of patients with full p16 expression in the recurrence group was 36%. There was no statistically significant correlation between the incidence of recurrence and the expression of p16 (*p* = 0.21872). Among 25 patients with recurrence 20 underwent reoperation; 5 patients were disqualified from surgery because of poor general condition.

In group of 20 patients after re-surgery the second recurrence occurred in 5 cases (25.0%); average time to relapse was 12 months.

## Discussion and conclusion

In scientific studies, using the query “inverted papilloma”; 1478 indexed publications were found, “inverted papilloma HPV” yielded 126 publications, and “Inverted papilloma p16” identified 19 publications.

The role of HPV infection in cervical cancer as well as cancers of the head and neck, esophagus, bladder, breast, and many others has been proven.^5^ The percentage of HPV + among patients with IP ranged between 38% and 63%.^4^, ^14^, ^15^, ^16^

Syrjänen et al. prepared a meta-analysis of research concerning HPV infection among patients with sinonasal papilloma (note that this is a broader concept than IP (includes fungiform, cylindrical (oncocytic) and inverted papilloma). They analyzed a total of 1956 cases. In total, 38.8% of cases were identified as HPV-positive (37.8% in the IP subgroup). Differences in the percentage of HPV-positive patients within different subgroups were explained by the different histological structure.^4^

The IP recurrence rate is estimated at 15%–20%.^17^, ^18^ Recurrent tumors tend to be more “aggressive”, with a greater tendency to further recurrences.^19^, ^20^ HPV infection seems to be a risk factor for recurrent IP.^21^

Expression of p16INK4a protein is considered to be the exponent of HPV infection, but there were reports to contradict this thesis. Yamashita et al. detected p16INK4a overexpression in only 1/4 of the HPV+ patients with squamous cell carcinoma, but in 80% of cases of HPV+ with IP, without showing a statistically significant correlation. The author concludes that p16INK4a cannot be considered a surrogate marker of HPV infection in the IP.^14^

Intraepithelial dysplasia is found in 5%–10% of inverted papillomas. There are no clear histological factors indicating the higher risk of malignant transformation; however, among the potential features, the presence of intraepithelial dysplasia (middle and high-grade) is mentioned, as is the presence of dyskeratosis and superficial keratosis in papilloma.^22^

The most common form of malignant transformation of inverted papilloma is squamous cell carcinoma, and less often is adenocarcinoma. Small cell cancer is a rare form of malignancy. Squamous cell carcinoma derived from IP is usually characterized by a high degree of differentiation, and thus have a better prognosis than other sinonasal squamous cell carcinomas. Even in the case of advanced changes, advanced lymphatic metastases are rare.^23^

Factors for malignant transformation of IP considered to be HPV infection; especially high risk HPV (HPV-16 or HPV-18),^5^, ^14^ smoking,^24^ p53 overexpression,^25^, ^26^ Ki-67 overexpression,^26^ cyclooxygenase-2 overexpression,^27^ a high degree of hyperkeratosis, high mitotic index, lack of inflammatory polyps and neutrophils, and the presence of plasma cells.^28^ It is believed that malignancy accompanies bilateral IP more often.^28^ Despite intensive research, certain predictors and biomarkers or malignancy have not yet been found.^23^ In the analyzed group, malignant transformation into squamous cell carcinoma occurred in 5 patients (one patient with full p16+ expression, two with borderline expression and two without p16 expression).

Zhao et al. prepared a meta-analysis of 31 studies exploring the relationship between HPV infection and malignant transformation of IP, stating that it is statistically significant, especially for HPV-18.^5^

The clinical features of IP will probably lead to further research that may allow the isolation of more perfect markers. A desirable feature would be not only defined group with an increased risk of IP, but also the identification of patients with already diagnosed IP with a higher risk of recurrence and malignant transformation. This is especially important when choosing treatment pathways as well as the regulation of post-operative follow-up.

## Conflicts of interest

The authors declare no conflicts of interest.
